# Effect of Plant Structure on Searching Strategy and Searching Efficiency of *Trichogramma turkestanica*

**DOI:** 10.1673/031.008.2801

**Published:** 2008-04-03

**Authors:** Daniel Gingras, Pierre Dutilleul, Guy Boivin

**Affiliations:** ^1^ AEF Global Inc. 201 Mgr Bourget, Lévis, Qc, Canada G6V 9V6; ^2^ Department of Plant Science, Macdonald Campus of McGill University, 21 111 Lakeshore road, Sainte-Anne-de-Bellevue, Qc, Canada H9X 3V9; ^3^ Horticultural Research and Development Centre, Agriculture and Agri-Food Canada 430 Boul. Gouin, St-Jean-sur-Richelieu, Qc, Canada J3B 3E6

**Keywords:** egg parasitoids, searching efficiency, searching strategy, host finding, movement, area searched, plant structure, activity, orthokinetic, klinotaxis

## Abstract

When searching for hosts on a plant, female parasitoids use strategies to maximize efficiency. Searching strategies include the expressed behaviors, the time budget associated with each behavior, the time allocated to the different plant parts and the exploration sequence of plant parts. Searching efficiency refers to the time taken to find the first egg, the number of eggs found per foraging time unit and the re-encountering frequency of eggs during a foraging period. This study examines the effect of artificial simple (few leaves and connections) and complex plant structures (more leaves and connections) on searching strategy and searching efficiency of the egg parasitoid *Trichogramma turkestanica* Meyer (Hymenoptera: Trichogrammatidae). Analyses of frequency and duration of behaviors associated with searching on artificial plants of different complexities were performed. Plant structure had no effect on time associated with locomotion behaviors such as walking, standing and flying. However, it had an impact on the area searched, which was significantly greater on simple plant structure. Also, time spent on a leaf without encountering an egg was greater on complex plant structure compared to simple one. No significant differences were found between simple and complex plant structures regarding time spent walking on the different plant parts such as twigs, limbs, leaf perimeters, and limbs of inferior and superior leaf sides. Results showed that female parasitoids spent less time actively exploring complex than simple plants. Encountering and re-encountering frequencies of eggs were significantly greater on simple than on complex plant structure. Plant structure had no effect on handling time of eggs. This study demonstrates that plant structure can modulate activities inherent to searching and ovipositing, which in turn affects area searched per foraging time unit and therefore host finding success.

## Introduction

When searching for hosts on a plant, foraging female parasitoids make strategic decisions such as where and how long to search and whether or not to accept a host once it is discovered. The outcome of these decisions can greatly influence the survival and fitness of parasitoids. Optimal foraging theory predicts that a forager should maximize its encounter rate with the most suitable and profitable hosts, and when hosts are abundant, avoid individuals of lower quality (Stephens and Krebs 1986). Searching strategies are implicit in many models that focus on parasitoid foraging behavior (Hassell and Southwood 1978; Vet and Dicke 1992; Weisser 1995; Powell et al. 1998; Vet 2001; Wajnberg 2006). Searching strategies result from genetic, learned, and sensory sources of information available to an insect and have been defined as sets of basic rules of scanning and movement that result in effective host encounter (Bell 1991; Vet and Dicke 1992).

Host encounter rate depends on the area searched per time unit (Skellam 1958), handling time, and reactive distance (Bruins et al. 1994). It also depends on the behavioral activities inherent to searching and ovipositing and the time budget of these activities. A female parasitoid that spends most of its time walking has more chance of finding a host than a sessile female. High walking velocity results in a higher probability of encountering hosts or cues that may lead the female to the host (Bieri et al. 1990 discussed in Bigler et al. 1997; Suverkropp et al. 2001). Bigler et al. (1988) showed a relationship between walking speed and parasitism of egg masses of *Ostrinia nubilalis* in the field by different laboratory-reared strains of *Trichogramma brassicae* (= *maidis*). Age of female parasitoids also appears to affect searching activity since young females, with a higher egg load, showed higher rates of movement than older ones with fewer eggs (Pak et al. 1985).

Most studies on foraging theory and foraging behavior have focused on host characteristics such as host density and distribution but the habitat, the plant and the plant structure may also affect parasitism levels (Romeis 2005). Plant structure can be defined by its size (height), heterogeneity (diversity of plant parts) and connectivity (absolute number of connections between plant components) (Gingras et al. 2002). Plant physical characteristics can impair movement, affect searching times and foraging success of predatory insects (Grevstad and Klepetka 1992) and parasitoids (Andow and Prokrym 1990; Lukianchuk and Smith 1997; Lovinger et al. 2000; Suverkropp et al. 2001; Wang and Keller 2001). Cloyd and Sadof (2000) found that different characteristics of plant structure such as plant height, size, leaf number, leaf surface area and branch number were negatively correlated with parasitoid attack rate.

In this study, searching strategies were defined as the time-budget of behavioral activities, the time allocated to different plant parts and the exploration order of plant parts. Searching efficiency includes the time taken to find the first egg, the number of eggs found per unit of foraging time and the re-encounter frequency of eggs during a foraging period. Based on the results of Gingras et al. (2002), who found that connectivity is the plant structure component that most affects foraging *Trichogramma*, we hypothesized that plant connectivity affects searching strategies and searching efficiency of female parasitoids. Female parasitoids may be more efficient when searching on simple plant structures than on complex ones. If linear movements are predominant, this hypothesis predicts that host eggs situated along the twig would be more frequently encountered and parasitized than those situated on leaves because they are located along a relatively straight line while those on leaves require that females take every connection met.

The present study examined, under laboratory conditions, the effect of plant connectivity on searching strategy and searching efficiency of *Trichogramma turkestanica* Meyer (Hymenoptera: Trichogrammatidae) foraging on simple and complex artificial plant structures containing eggs of the host *Ephestia kuehniella* (Zeller) (Lepidoptera: Pyralidae).

## Materials and Methods

All experiments used female *T. turkestanica* reared at 24° C, 16:8 L:D, on cold-killed eggs of the Mediterranean flour moth, *E. kuehniella*. Female parasitoids were less than 6 hours old, mated, unfed prior to the experiment, naive with respect to the plant and had no previous oviposition experience.

**Figure 1.**
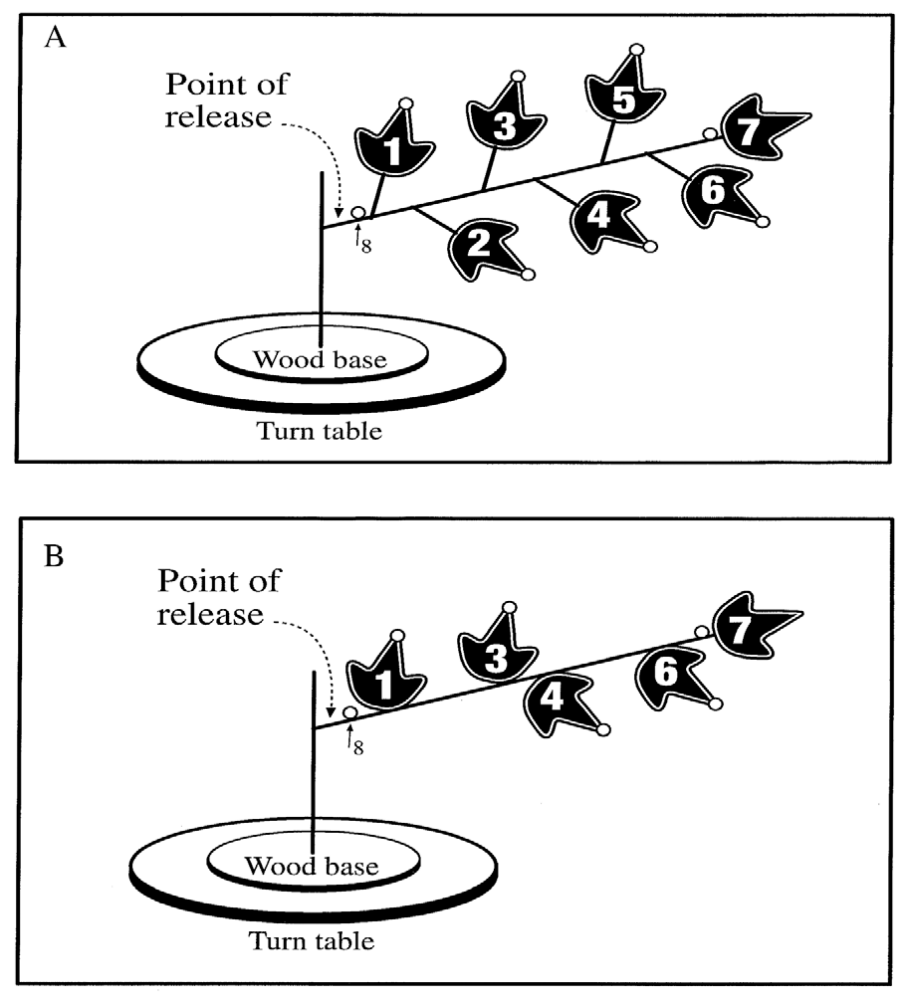
Representation of the experimental set-up for the complex (A) and simple (B) artificial plant structures. The complex plant consisted of a plastic twig 20 cm long comprising 7 leaves and 6 petioles 1.5 cm long. The twig was supported by a straight shaft 5 cm long inserted in a wood base. The simple plant was identical to the complex one, except leaves number 2 and 5 and all petioles were removed. The white circles represent the eggs. The digits on leaves correspond to leaf and egg numbers. The point of release of *Trichogramma* females is also represented.

Plant structure can be defined by its size (height), heterogeneity (diversity of plant components) and connectivity (absolute number of connections between plant components) (Gingras et al. 2002). Simple and complex plants were used to detect an effect of plant structure on rates of parasitism. The complex artificial plant consisted of a plastic twig 20 cm long, comprising 7 leaves and 6 connections (petioles) of 1.5 cm long. The twig was supported by a straight 5 cm long segment, which was inserted in a wood base (Figure 1 a). To obtain the simple artificial plant, all connections were cut and leaves number 2 and 5 were removed. Thus, simple plants had no connections and only 5 leaves that were directly glued to the twig (Figure 1 b). On each leaf and on both sides (inferior and superior), a limb (in black in Figure 1) and a perimeter, a zone situated within 3 mm of leaf margin, represented in white on Figure 1, were distinguished. On the inferior side, leaves had veins. One egg of *E. kuehniella* was glued at the tip of each leaf, within the perimeter zone on the superior side, and two eggs were glued on the twig, one at each extremity (Figure 1). The digit on the leaf was also used to identify eggs (leaf1, egg1, etc...); egg number eight was closest to the release point, on the twig. This experiment controls for changes in egg density as it was close to 0.13 egg/cm2 for both simple and complex plants.

The experiment took place at 25 ± 1° C within a cage (85 × 85 × 100 cm) covered with white muslin. A turntable supported the experimental set-up and permitted continuous observations of the parasitoid. In addition to the fluorescent light in the room, a fluorescent light, situated at a height of 45 cm over the plant lit the experimental arena and created a light intensity of 1.05 kilolux on the plant. The light intensity was measured with a Li-Cor (www.licor.com), model Li-1000-32 data logger apparatus.

One female *Trichogramma* was released on the artificial plant (Figure 1) and was monitored by direct observation with the naked eye, for one hour or until it left the plant. Replicates of less than 20 minutes, and those where the parasitoid was lost to sight for more than one minute, were discarded. A new twig was used for each of the 30 replicates per plant structure. The information relative to movements of the female on the plant was classified into four categories: locomotion, substrate, leaf side and leaf number. Locomotion behaviors included walking, standing, and flying. The substrate categories were the twig, limb, perimeter, veins and eggs. The leaf sides were either inferior or superior. Finally, the leaf numbers were coded 1 to 7 for the complex plant and 1, 3, 4, 6 and 7 for the simple plant. Observations on searching behavior of the female were recorded on a portable Tandy 1000 compatible IBM computer, using Observer software, version 2.0 (Noldus Information Technology 1991, www.noldus.com).

### Data treatment and statistical analysis

The area searched by female parasitoids was estimated by using the formula S/A = N_a_/N (Wiedenmann and O'Neil 1992), where S is the estimate of leaf area searched (in cm^2^), A is the total amount of leaf area available to search (simple: 43 cm^2^; complex: 60 cm^2^), Na is the number of hosts encountered, and N is the number of hosts available for parasitization (6 for simple, 8 for complex plants).

Encountering frequency corresponded to the number of times an egg was encountered by a female between the replicates whereas re-encountering frequency corresponded to the number of times a female came back to an egg previously encountered within the same replicate. Mean and cumulative values of encountering frequency and re-encountering frequency were computed over replicates where at least one egg was encountered by females (n = 23 for simple plants, n = 11 for complex plants). Time elapsed between successive discoveries of two different eggs was estimated from replicates where the female encountered at least two different eggs within one replicate (n = 11 for simple plants, n = 1 for complex plants). No statistical test was performed in the latter case because of lack of replicates for complex plants.

*t* tests for comparison of two means, with preliminary assessment of the equality of variances (*F* tests), were used to determine if plant structure had an overall effect on searching strategies and searching efficiency. Because of inequalities in the length of the observational period depending on the replicate or the group (simple vs. complex plants), proportions were used in statistical analyses. When the variable was a duration of activity, a proportion was calculated as the ratio of the initial value of the variable to the length of the corresponding observational period. Prior to the *F* and *t* tests, the proportion data were submitted to the arcsine-square root transformation (Snedecor and Cochran 1967). Substrate and leaf side categories were combined to refine the analysis of movement of female parasitoids on the plant. Extra categories were: 1) limb inferior side, 2) limb superior side, 3) within-perimeter inferior side, 4) within-perimeter superior side. Statistical tests were performed with SAS procedure TTEST (SAS Institute 2004).

To test if plant structure influenced residence time, the proportion of females that stayed for one hour were compared between plant structures. To test if plant structure influenced flight and leaving frequencies, a 2 × 2 contingency table was constructed. Differences were compared under the null hypothesis of equal frequencies between simple and complex plant structure by using a Chi-square test.

## Results

The foraging behavior that was typically observed starting from the release point can be described as walking along the twig, taking a connection (a petiole), walking rapidly along the latter, exploring the leaf by walking in a relatively straight course, and walking interrupted by sharp turns. Such observations were also reported by Gardner and van Lenteren (1986). Females often stayed for long periods not moving and preening themselves.

### Searching strategy

Plant structure had no significant effect on duration of activities related to locomotion such as walking, standing and flying (Table 1). Flying rarely occurred and when observed, it was to leave the plant. Flying was observed in 13 replicates for both simple and complex plants.

The time spent on the different plant parts did not differ between simple and complex plants, except for the veins (Table 1). Female parasitoids spent significantly more time on veins when on a complex plant compared to a simple one. When comparing the time budget for the inferior and superior sides of leaves, no significant difference was found for the side of the leaf and the limb (Table 1). However females spent significantly more time on the inferior side of the perimeter of complex plant compared to simple plant.

**Table 1.**
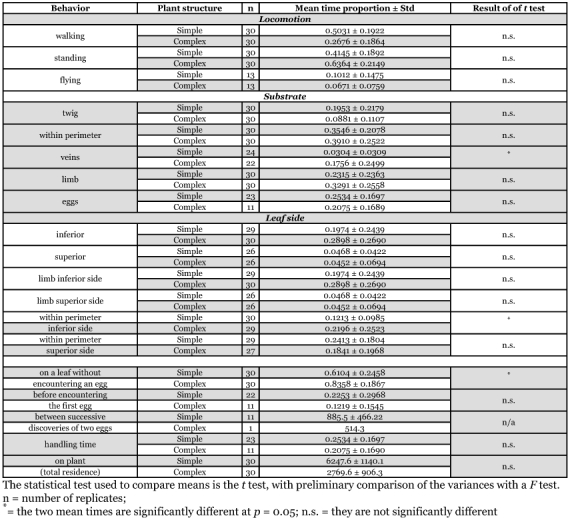
Comparison of means between the two experimental groups for variables related to the time spent by *Trichogramma* females in various activities and expressed as a proportion (i.e., relatively to the length of the observational period).

### Searching efficiency

Time spent on leaves without encountering an egg was significantly lower on simple plant structures than on complex ones (Table 1). The average time to encounter the first egg and handling time were not influenced by plant structure. The time elapsed between successive discoveries of two different eggs may have been higher on the simple plant structure compared to the complex plant structure, but it was observed in only one replicate on the complex plant. The average number of different eggs found per hour, which reflects host finding capacity and searching efficiency at the scale studied was 2.13 (or 1.19 ± 0.35 after arcsin transformation; n = 23) on simple and 1.09 (or 0.95 ± 0.09 after arcsin transformation; n = 11) on complex plants and a t-test showed significant differences (t value = -2.43; P< 0.005). The mean proportion of area searched by female parasitoids was almost twice as large on simple (0.36 ± 0.17 cm^2^; n = 23) than on complex (0.18 ± 0.08 cm^2^; n = 11) plants and this difference was significantly different (t-value = -3.35; P< 0.005). Plant structure had no effect on residence time on plants by female parasitoids. The patch residence time of females was not statistically significant (χ^2^ = 0.62, NS).

All eggs were encountered on simple plant structures but not on complex ones as revealed by encountering frequency values (Table 2). On both simple and complex plant structures, egg 7 was the least frequently encountered despite being situated on the twig, thus along a relatively straight line trajectory from the point of release. Females had a strong tendency to come back to an egg previously encountered as indicated by high cumulative and mean re-encountering frequency values. Cumulative and mean re-encountering frequencies of an egg were significantly higher on simple than on complex plant structure for egg situated on leaf number 1, 3, 4, and 6. Also, a female came back between two and four times to the same egg on simple plant structure and four times for the only replicate where it occurred on complex plant structure (Table 2).

**Table 2.**
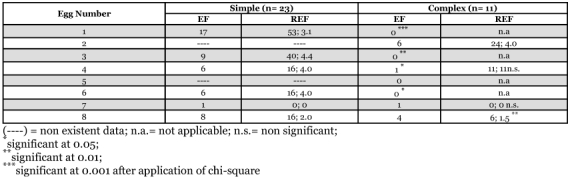
Cumulative encountering frequency (EF) and cumulative and mean re-encountering frequency (REF) of eggs for replicates where at least one egg was found.

## Discussion

Searching within the host habitat in the absence of cues from hosts or plants is assumed to be random for most parasitoids (Waage 1979; Vinson 1984). In this experiment, it was assumed that females did not have access to cues as plastic plants and dead host eggs were used. In such a context, the number of eggs encountered will depend on the area searched per time unit (Skellam 1958). Indeed, on the simple plant structure, females explored greater surface areas per foraging time unit and found host eggs at a higher frequency.

However, searching has a cost as it reduces time allocated to oviposition and eating and increases risks associated with predation (Stephens and Krebs 1986). Female parasitoids should aim to increase the area searched per time unit through the use of efficient searching strategies in order to encounter greater number of hosts and have greater fitness.

### Searching strategy

The area searched per unit time depends on the type of activity and the time budget of these activities. For sedentary hosts, like eggs, female parasitoids that adapt a strategy that maximizes movement, either by walking or flying to cover most of the structure of a plant should be more adaptive. Flight was not observed frequently and when it was observed, it was to leave the plant but was rarely used to move from one plant part to another. Yet, dispersal by female *Trichogramma* to other plants or plant parts increases their chances of finding hosts or host cues (Bell 1991). Observations on flight by *Trichogramma* are rare (reviewed in Keller et al. 1985; Noldus et al. 1988; Forsse et al. 1992), and understandably so because the minute size of the insect makes it difficult to observe. *Trichogramma* females covered most plant parts but, while on leaves, spent one third of their time on a limb, a result that can partially explain the low encountering frequency of eggs since the latter were located around the perimeter. Such a result is different from the observations of Suverkropp (1997), who observed that *T. brassicae* (= *maidis*) females spent 18 to 24 percent of their time on the leaf following veins and edges and those of Noldus et al. (1991) who observed that *T. evanescens* spent a significant amount of time on the leaf edge of Brussels sprouts. Moreover, female *T. turkestanica* spent, whatever the plant structure was, a greater amount of time on the inferior rather than on superior side of leaves. *Trichogramma brassicae* also spent a greater proportion of time on lower rather than upper surface of corn leaves (Gass 1988 discussed in Bigler et al. 1997).

### Searching efficiency

Searching efficiency is related to host finding capacity, which is defined by the number of hosts found per unit of time (Vet 2001). At the plant scale studied, female parasitoids found almost twice as many eggs on simple (2.13 per hour) than on complex plant structures (1.09 per hour). Females encountered eggs more frequently on simple plants because the spatial organization of these may have favored linear walking and high walking velocity which increase probability of encountering hosts or cues (Bieri et al. 1990 in Bigler et al. 1997). Any increase in connectivity also increases the number of pathways and possible directions that a foraging female can take to find an egg. Females may stop more frequently or reduce walking speed prior to taking one pathway or another. Thus, orthokinetic movements become more difficult and overlap of search paths may occur more frequently on complex plant structures. For identical host densities per plant, such as in this study, parasitoids may perceive complex plants as patches of lower quality because more time is needed to discover hosts. However, mean proportion of total residence time on the plant was not affected by plant structure. One hypothesis for such a result could be the presence of a lower threshold of complexity over which such differences could be observed in total residence time. Factors that influence patch residence time include richness and quality of the patch, probability of survival of the female and travel risks (Stephens and Krebs 1986; Bell 1991). On simple plant structures, richness and quality decreased more rapidly, which may explain why females left more frequently before the hour of observation was completed.

It was expected that eggs situated along the twig (eggs 7 and 8), thus along a straight line, would be more frequently encountered due to orthokinetic movement predominance. As revealed by the observed frequencies of leaves explored, klinotaxis movements were predominant, which explains why leaves close to the releasing point were visited first. Such behavior also explained why egg 7, either on simple or on complex plants was rarely encountered. It was expected that time before encountering the first egg would have been influenced by plant structure. The similar spatial configuration and egg position for the simple and complex plant models and the searching strategy consisting in exploring the first connection met could explain why no differences were obtained.

After ovipositing, parasitoids, including *T. turkestanica*, often make an intensive investigation of the area adjacent to the host, an observation reported in previous studies (Laing 1937; Jackson 1966; Hokyo and Kiritani 1966). Moreover, females had a strong tendency to come back more than once to the egg that they had just parasitized, a behavior that reduced searching efficiency and that may lead to superparasitism. What could be the benefits of such a behavior? It is possible that it is more common with younger and unexperienced females. Typical egg laying behavior was sometimes observed on a previously parasitized egg, suggesting superparasitism. Rosenheim and Mangel (1994) report that a parasitoid with an imperfect ability to discriminate between unparasitized hosts and hosts that it had attacked earlier within the same patch, experiences a risk of self-superparasitism when attacking multiple hosts within a single patch. Repeating these experiments using females of different ages and levels of experience relatively to egg laying may answer these questions.

Complexity of plants is an important factor in foraging activities. In nature, few host species are found on a uniform, featureless surface, and parasitoids going through the host location process will often require more than a single strategic decision. The discovery of a host represents the outcome of a hierarchical series of decisions that impact the capacity of parasitoids to maximize their lifetime fitness gain.
